# Altered Cerebral Cortical Gyrification in Ferrets with Neonatal Exposure to the Bacterial Endotoxin, Lipopolysaccharide

**DOI:** 10.1523/ENEURO.0135-25.2025

**Published:** 2025-07-31

**Authors:** Kazuhiko Sawada, Rie Ryoke, Hiroi Nonaka, Ryuta Kawashima, Akira Sumiyoshi

**Affiliations:** ^1^ Department of Nutrition, Faculty of Medical and Health Sciences, Tsukuba International University, Tsuchiura, Ibaraki 300-0052, Japan; ^2^ Institute of Development, Aging and Cancer, Tohoku University, Sendai, Miyagi 980-8575, Japan; ^3^ National Institutes for Quantum Science and Technology, Chiba 263-8555, Japan

**Keywords:** cerebral cortex, ferret, gyrification, lipopolysaccharide, maternal immune activation

## Abstract

Lipopolysaccharide (LPS) is a bacterial endotoxin that induces innate immune responses. The present study aimed to elucidate alterations in cerebral cortical surface morphology induced by neonatal exposure to LPS using gyrencephalic ferrets. Male ferret pups received a subcutaneous injection of LPS (500 µg/g of body weight) on Postnatal Day (P)6 and P7. Furthermore, EdU and BrdU were administered on P5 and P7, respectively, to label postproliferative and proliferating cells that were exposed to LPS in the late stage of cortical neurogenesis. On P20 when the primary sulci and gyri had formed, MRI-based morphometry revealed an anterior shift in sulcal infolding in the medial and dorsolateral cortices of LPS-exposed ferrets. Immunofluorescence analysis showed that LPS increased the density of BrdU-labeled cells and reduced their apoptosis, as indicated by cleaved caspase-3 (cCasp3) immunostaining, in the outer stratum of the lateral sulcus located on the parietal association cortex. Furthermore, cCasp3 immunostaining of EdU-labeled cells was enhanced in the presylvian and lateral sulci located in the prefrontal and parietal association cortices, respectively, but was reduced in the coronal sulcus and gyrus located on the primary motor cortex in LPS-exposed ferrets. This study is the first to elucidate the effect of bacterial components on cerebral cortical sulcogyrogenesis, which is involved in the pathogenesis of neurodevelopmental disorders. Such altered sulcal topology may be attributed to a region-related effect on cortical neuron apoptosis in the medial and dorsolateral cortices caused by neonatal LPS exposure.

## Significance Statement

The present study aimed to elucidate alterations in cerebral cortical surface morphology of gyrencephalic ferrets caused by neonatal exposure to lipopolysaccharide (LPS), a bacterial endotoxin. The observed anterior shift in sulcal infolding in the medial and dorsolateral cortices of LPS-exposed ferrets provides the first evidence of the effects of bacterial components on cerebral cortical sulcogyrogenesis, which is known to be involved in the pathogenesis of neurodevelopmental disorders such as autism spectrum disorders and schizophrenia. Such altered sulcal topology may be attributed to a region-related effect on cortical neuron apoptosis in the medial and dorsolateral cortices induced by neonatal LPS exposure.

## Introduction

Innate immune activation triggered by bacterial and viral infections during pregnancy alters fetal neurodevelopment, increasing the risk of neurodevelopmental disorders such as autism spectrum disorder (ASD), attention-deficit/hyperactivity disorder, and cognitive dysfunction (Knuesel et al., 2014; Estes and McAllister, 2016; Kwon et al., 2022; Shook et al., 2022); this phenomenon is termed maternal immune activation (MIA; Knuesel et al., 2014; Kwon et al., 2022). Many studies have documented brain abnormalities in MIA using lissencephalic animal models such as rodents (Wischhof et al., 2015; Crum et al., 2017; Guma et al., 2019; Aria et al., 2020; Lee et al., 2021; Vojtechova et al., 2021). In contrast, bacterial components have been reported to directly influence cerebral cortical development, triggering abnormal cognitive behaviors, via toll-like receptors independent of MIA (Humann et al., 2016; Mann et al., 2023). Lipopolysaccharide (LPS), an endotoxin of gram-negative bacteria, triggers innate immune activation, as do peptidoglycan, β-glycan, and polyinosinic:polycytidylic acid (Poly I:C). LPS directly acts on neural stem cells/progenitors by activating toll-like receptor 4 (TLR4), regulating their proliferation and differentiation, and preventing their apoptosis (Grasselli et al., 2018). LPS promotes the proliferation, but not apoptosis, of subventricular zone (SVZ) progenitors, including intermediate progenitors (IPs; Pang et al., 2016; Sawada et al., 2023), which are considered major sources of the cerebral cortical expansion (Kelava et al., 2012).

Gyrification abnormalities have been reported in human patients with neurodevelopmental disorders such as ASD and schizophrenia, with phenotypes varying depending on the disorder and patient's sex (Schaer et al., 2013; Wallace et al., 2013; Nenadic et al., 2015; Matsuda and Ohi, 2018; Rafiee et al., 2022; Howes et al., 2023). Ferrets are gyrencephalic animals that experience the late stage of corticoneurogenesis and begin cortical sulcogyrogenesis during the first postnatal week (Kelava et al., 2012; Martínez-Cerdeño et al., 2012; Sawada and Watanabe, 2012; Sawada and Aoki, 2017), corresponding to the neurodevelopmental stage of the cerebral cortex in primates at midgestation. Recently, abnormal sulcal infolding has been reported in association cortical regions of neonatally valproic acid (VPA)-exposed ferrets (Sawada et al., 2021), which display ASD-like social behavior impairments (Krahe et al., 2016). This abnormal sulcal infolding may be attributed to VPA's effect on promoting proliferation and neural differentiation (differentiative division) of SVZ progenitors (Sawada, 2022). These findings led to the hypothesis that the differentiative division of SVZ progenitors induced by LPS exposure (Sawada et al., 2023) results in gyrification abnormalities in the ferret cortex. The present study aimed to elucidate alterations in cerebral cortical surface morphology caused by neonatal LPS exposure in ferrets using MRI morphometry. Herein, we tracked SVZ progenitors exposed to LPS in postproliferative and proliferating states to determine their fate and to explore their relationship with sulcogyrogenesis.

## Materials and Methods

### Animals

This study used eight male ferrets, naturally delivered by five pregnant ferrets, which were purchased from Japan SLC (Hamamatsu). These ferrets were reared with lactating dams (3–5 pups/dam) in stainless-steel cages (80 × 50 × 35 cm) maintained at 21.5 ± 2.5°C under 12 h of artificial illumination in the Facility of Animal Breeding, Nakaizu Laboratory, Japan SLC. All dams were fed a pellet diet (High-Density Ferret Diet 5L14; PMI Feeds) and had access to tap water *ad libitum*. This study was approved by the Institutional Animal Care and Use Committee of Tsukuba International University (Approval Code, 30-1; December 4, 2018). All efforts were made to minimize both the number of animals used and their suffering.

The administration schedule for LPS and thymidine analogs was described in a previous study (Sawada et al., 2023). Eight male ferrets received intraperitoneal injections of 5-ethynyl-2′-deoxyuridine (EdU) at 30 µg/g body weight (Sigma-Aldrich) on Postnatal Day (P)5 and 5-bromo-2′-deoxyuridine (BrdU) at 30 µg/g body weight (Sigma-Aldrich) on P7. LPS was administered to four ferrets at 500 µg/g body weight on P6 and P7. The second LPS injection was given concurrently with the BrdU injection. When EdU and BrdU were administered following this schedule, cells that proliferated 24 h prior to LSP exposure were labeled with EdU, while cells that proliferated immediately after LPS exposure were labeled BrdU. The remaining four ferrets that did not receive LPS injections were used as controls. All ferrets were reared until P20, when the primary sulci and gyri had formed (Sawada and Watanabe, 2012; Sawada and Aoki, 2017). Subsequently, all ferrets were perfused with 4% paraformaldehyde in PBS under deep anesthesia with ∼2% isoflurane gas. The body weight on P20 was 97.4 ± 15.3 g in the LPS-exposed group (*n* = 4) and 110.2 ± 10.1 g in the control group (*n* = 4). The brain weight was 3,410 ± 395 mg in the LPS-exposed group (*n* = 4) and 3,730 ± 264 mg in the control group (*n* = 4). No significant difference in brain weights between the two groups was detected using Student's *t* test (*p* = 0.416).

### MRI measurements

Three-dimensional anatomical images were acquired with a 7.0 T MRI system (Pharmascan 70/16, Bruker BioSpin) using a 38-mm-diameter birdcage coil. The magnetization-prepared rapid gradient-echo sequence was employed with the following parameters: TR, 10.93 ms; TE, 3.20 ms; inversion delay, 1,200 ms; segment repetition time, 6,000 ms; segment duration, 2,798 ms; averages, 40; FOV, 25.6 × 25.6 × 32.0 mm^3^; matrix, 256 × 256 × 320; voxel size, 0.1 × 0.1 × 0.1 mm^3^. The total scanning time was 24 h.

### MRI-based morphometry

Cerebral cortical volume, cortical surface area, and fronto-occipital (FO) length were measured on coronal (transaxial) MR images at equal *z*-axis intervals (100 µm) using the SliceOmatic software v4.3 (TomoVision), as described in previous reports (Sawada and Aoki, 2017; Sawada et al., 2021). The cerebral cortex was broadly divided into four regions: the prefrontal, frontal, temporoparietal, and occipital. The boundaries of these regions were defined based on structural landmarks on coronal MR images, following a previously published method (Sawada et al., 2015). The cortical volume and surface area were estimated for each of these four cortical regions.

To measure the cortical thickness, the cerebral cortex was semiautomatically segmented based on signal contrasts and was rendered in 3D using the SliceOmatic software. The resulting 3D image was then used to compute the mean cortical thickness across the entire cerebral hemisphere using Amira v5.2 (Visage Imaging), as previously described (Sawada and Aoki, 2017; Sawada et al., 2021).

### Gyrification index (GI)

GI values were measured using the SliceOmatic software (TomoVision), as previously described (Sawada and Aoki, 2017; Sawada et al., 2021). Briefly, the cortical outer contours and perimeters of each sulcus were computed separately from the coronal MR images. The ratio of the length of the outer contour to the sum of the sulcal perimeters was calculated for all images, and the mean of these values was defined as the global-GI. The sulcal-GI was determined as the mean of the contour-to-perimeter ratios for seven primary sulci, using the same method. The regional-GI was further estimated as the mean value in the four cortical regions defined previously.

The rostrocaudal GI distribution was mapped using the SliceOmatic software (TomoVision), as previously described (Sawada and Aoki, 2017; Sawada et al., 2021). Briefly, the inner and outer cortical contours were computed on coronal MR images at equal *z*-axis intervals of 100 µm. The GI for each coronal slice was calculated as the ratio of the outer contour length to the inner contour length. The mean GI was estimated for each slice, and the rostrocaudal distribution of GI across the cerebral cortex was visualized by defining the coronal slice at the level of the anterior commissure as “slice number 0.”

### Immunohistochemical procedures

Cerebral hemispheres were immersed in a 30% sucrose–PBS solution overnight and then embedded in an optimal cutting temperature compound (Sakura Finetek) at −70°C. Coronal cryosections of the hemispheres at 100 µm thickness were made using a Retratome (REM-700; Yamato Koki Industrial) equipped with a refrigeration unit (Electro Freeze MC-802A; Yamato Koki Industrial).

**Figure 1.**
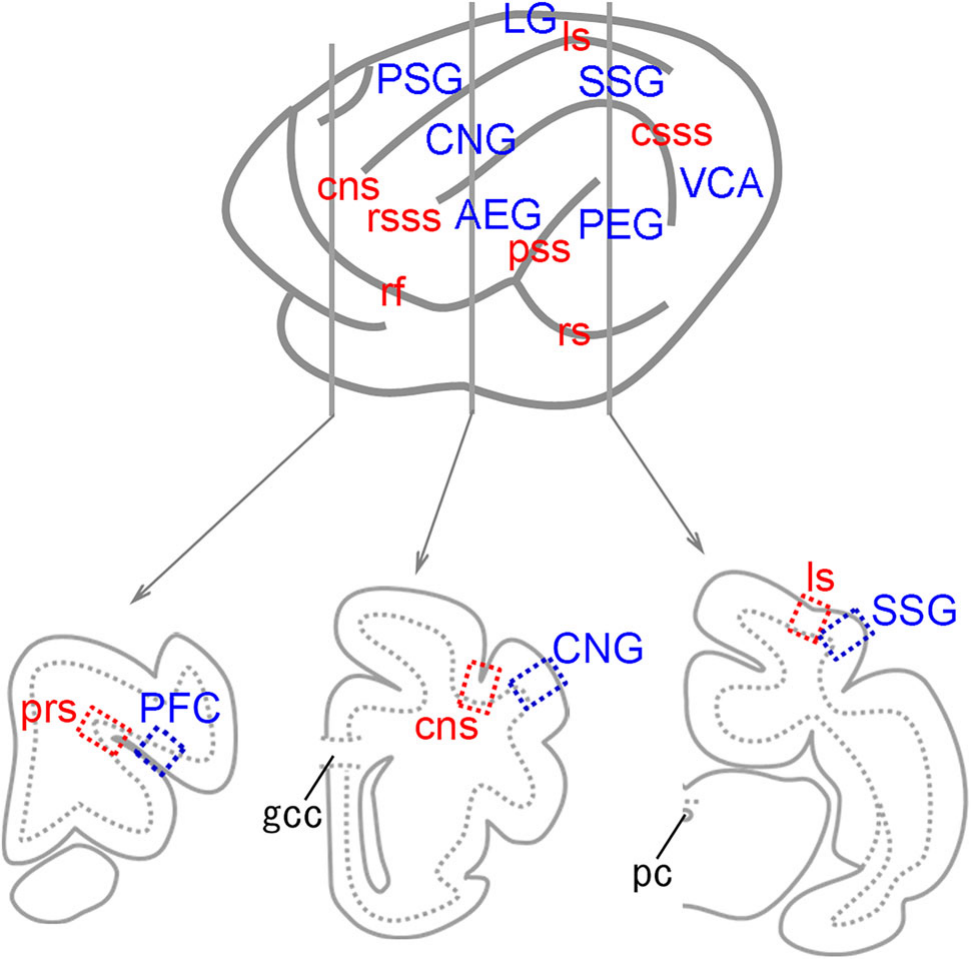
Positions for making coronal sections for immunostaining with thymidine analog labeling. Illustrations of lateral surfaces of the left cerebral hemisphere in 20-d-old ferrets and coronal images at positions for making histological sections are shown. Blue- and red-dotted rectangles indicate the positions of gyral crowns and sulcal floors, respectively, for capturing immunofluorescent images for stereological analysis. CNG, coronal gyrus; cns, coronal sulcus; csss, caudal suprasylvian sulcus; gcc, genu of the corpus callosum; LG, lateral gyrus; ls, lateral sulcus; pc, posterior commissure; PFC, prefrontal cortex; PEG, posterior ectosylvian gyrus; prs, presylvian sulcus; PSG, posterior sigmoid gyrus; pss, pseudosylvian sulcus; rf, rhinal fissure; rs rhinal sulcus; rsss, rostral suprasylvian sulcus; SSG, suprasylvian gyrus; VCA, visual cortical area.

**Figure 2.**
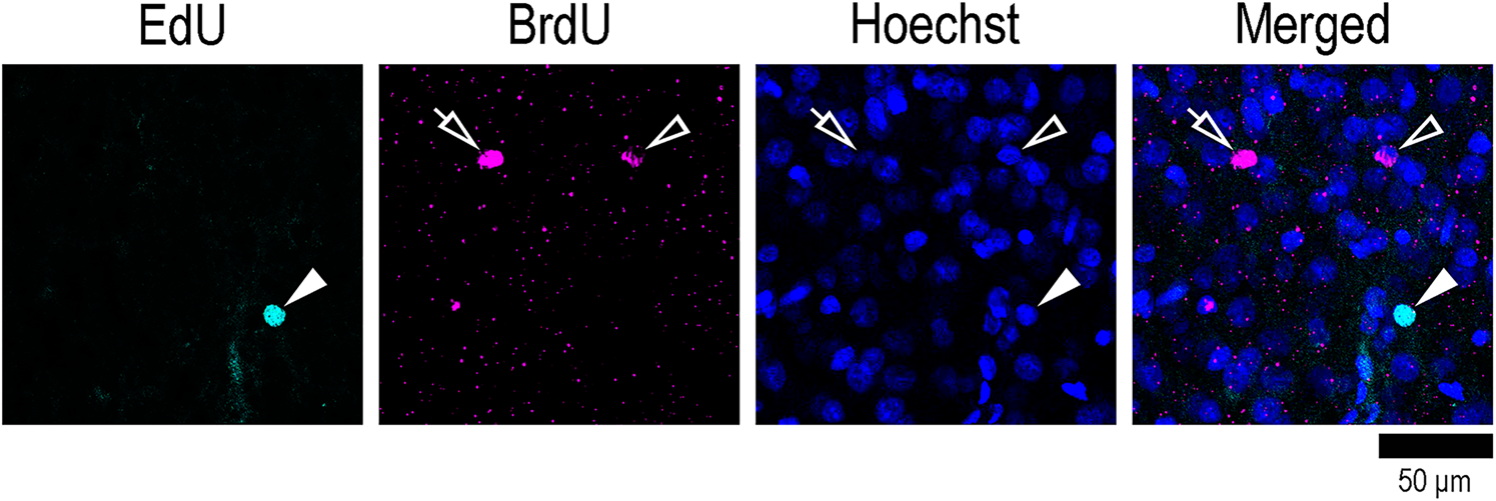
High-magnification images of EdU- and BrdU-single–labeled cells with Hoechst staining in the OS of the cortical crown of the CNG. Closed arrowheads indicate EdU labeling. Open arrows and open arrowheads indicate BrdU labeling. Both EdU and BrdU labeling are overlayed with Hoechst staining. In some cases, Hoechst staining was weak, when BrdU labeling appeared was clear (open allows). BrdU, 5-bromo-2′-deoxyuridine; EdU, 5-ethynyl-2′-deoxyuridine.

Three sets of serial coronal sections taken around the levels shown in Figure 1 were subjected to immunofluorescence and EdU staining. These sections included the representative primary sulci and gyri in the prefrontal cortex [presylvian sulcus (prs) and prefrontal cortex (PFC)], primary motor cortex [coronal sulcus (cns) and coronal gyrus (CNG)], and rostral posterior parietal cortex [lateral sulcus (ls) and suprasylvian gyrus (SSG); Fig. 1]. All procedures were performed on free-floating sections. Sections were heated in Antigen Unmasking Solution H-3300, pH 6.0 (Vector Labs), at 90°C for 30 min in a water bath and then cooled at 4°C for 30 min. After preincubation in PBS containing 0.1% Triton X-100 (Triton-PBS) at 37°C for 1 h, EdU detection was performed using the Click-iT reaction cocktail (Click-iT EdU Alexa Fluor 488 Imaging Kit, Thermo Fisher Scientific). Immunofluorescence staining was performed using the primary antibodies listed in Table 1. The primary antibodies used were highly specific to the brain tissue (Horiuchi-Hirose and Sawada, 2016; Sawada and Kamiya, 2023; Sawada et al., 2023). The sections were then incubated with a mixture of Alexa Fluor 555-labeled donkey anti-rabbit IgG (1:500; A-31572, Thermo Fisher Scientific) and Alexa Fluor 647-labeled donkey anti-sheep IgG (1:500; ab150179, Abcam) as secondary antibodies at 37°C for 2 h. Some sections were counterstained with NeuroTrace 640/660 (Nissl stain; N21483, Thermo Fisher Scientific) to confirm that EdU and BrdU labeling were localized in the cellular nuclei. After washing with PBS, the sections were mounted on slides and coverslipped with glycerin. We also confirmed that EdU and BrdU labeling overlapped with the nuclear marker, Hoechst 33342 (Thermo Fisher Scientific; Fig. 2). We used the terms “immunopositive” or “positive labeling” (+) to refer to cells exhibiting high to moderate levels of marker antigens or thymidine analogs and “immunonegative” or “no labeling” (−) for cells with low or no detectable labeling.

**Table 1.** Primary antibodies used in the study

Antigens	Sources	Species	Catalog #	RRID	Concentration used	References
BrdU	Abcam	Sheep polyclonal	ab1893	AB_302659	1:1,000	Sawada et al., (2023) Int J Mol Sci
NeuN	Millipore	Rabbit polyclonal	ABN78	AB_10807945	1:500	Horiuchi-Hirose and Sawada (2016) Anat Rec
S100	ImmunoStar	Rabbit polyclonal	942,001	AB_572261	1:500	Sawada and Kamiya (2023) Congenit Anom
cCasp3	GeneTex	Rabbit polyclonal	GTX22302	AB_384753	1:500	Sawada et al., (2023) Int J Mol Sci

### Estimation of cell density

Serial digital sectioning images (10 sections at 10 µm intervals in depth) were captured under a 20× objective using an Axio Imager M2 ApoTome.2 microscope equipped with an AxioCam MRm camera (Carl Zeiss) with the Zen 2.3 Blue Edition software (Carl Zeiss). The densities of immuno- and thymidine analog-labeled cells were calculated by the disector method with systematic random sampling, as described previously (Sawada et al., 2021). To estimate cell density, we selected a set of sectional images, 4 µm apart in the *Z*-direction (the third and seventh slices from the superficial slices of the captured image sets), as the reference and lookup images, respectively. Frames with 48 square boxes (box size, 40 × 40 µm) were used to systematically select the region of interest (ROI) superimposed randomly on the outer stratum (OS; Layers II–III) and inner stratum (IS; Layers IV–VI) of the sulcal floors and gyral crowns. The proportions of immunostained and/or thymidine analog-labeled cells were estimated by summing the cells counted within the ROIs.

### Statistical analysis

Measurements from the left and right hemispheres were pooled, and the number of animals (*n*) was set to four per group. The densities of immuno- and thymidine analog-labeled cells were statistically analyzed using repeated-measure two–way ANOVA, with “region” (OS and IS) and “group” (LPS-exposed and control groups) as factors. Scheffé's test was used for post hoc comparisons to detect significant effects on groups and/or region × group interactions using repeated-measure two–way ANOVA. The proportion of cells immunoreactive for marker antigens among thymidine analog-labeled cells was assessed using the *χ*^2^ test. The total number of EdU-single- and BrdU-single–labeled cells was defined as “*n*” for the *χ*^2^ test.

## Results

### MRI-based morphometry

**Figure 3.**
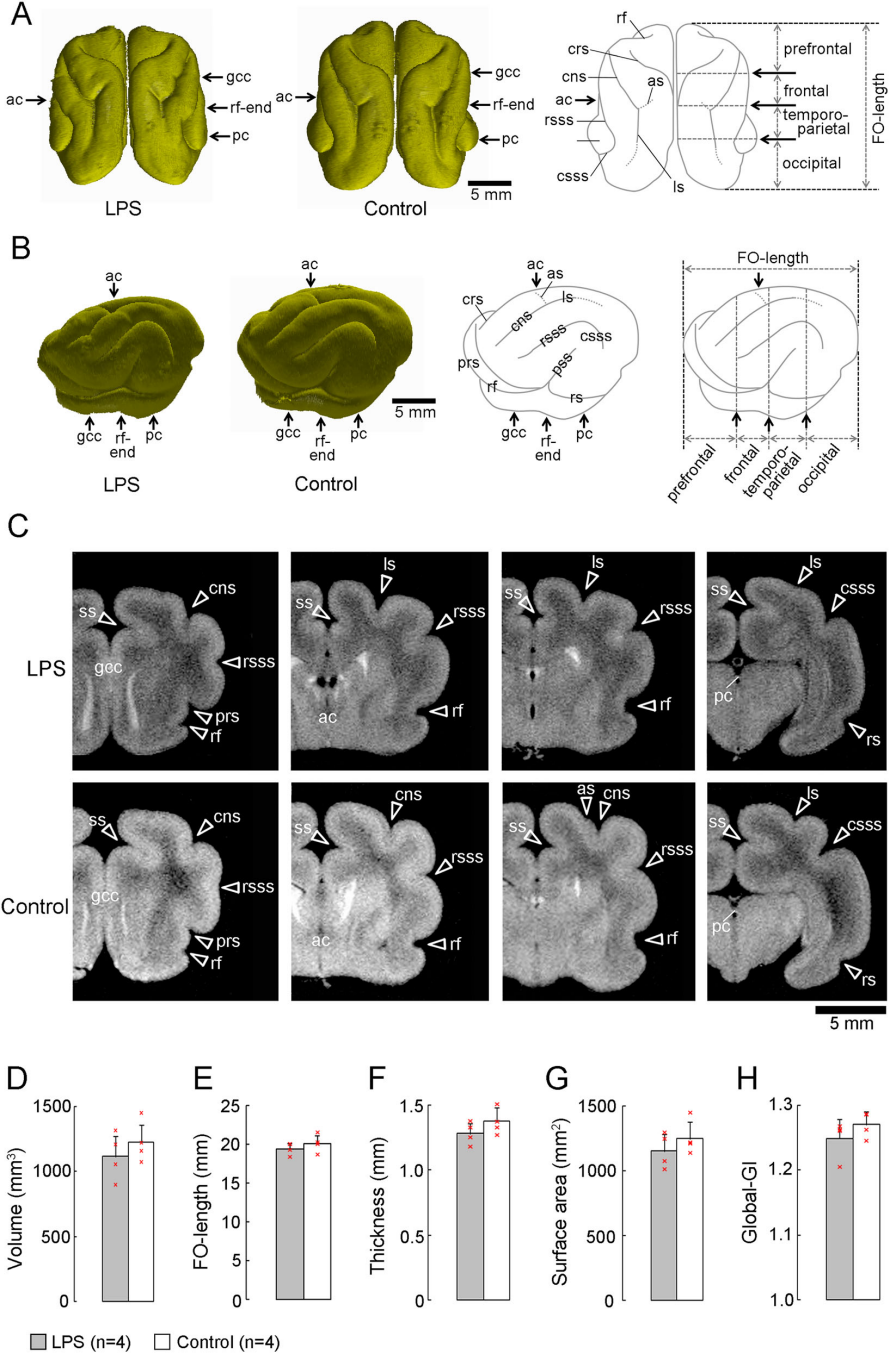
MRI-defined morphological aspects of the cerebral cortex. ***A***, Three-dimensional–rendered images of the cerebral hemispheres (dorsal view) for LPS-exposed (left) and control (center) ferrets. An illustration of the dorsal surfaces of the cerebral hemispheres indicates primary sulci (right), the boundaries of four subdivisions of the cerebral cortex (right), and the reference for measurements of FO-length (right). ***B***, Three-dimensional–rendered images of the left hemispheres, lateral view, for LPS-exposed (left) and control (second from the left) ferrets. Illustrations of the lateral cerebral surface of the cerebral hemisphere indicate primary sulci (second from the right), the boundaries of four subdivisions of the cerebral cortex, and the reference for measurements of FO-length (right). ***C***, Three-dimensional anatomical MR images of the cerebrum at the coronal plane from LPS-exposed and control ferrets. Coronal MR images are shown from left to right in the following order: the prefrontal region at the rostral end of the genu of the corpus callosum (gcc), frontal region at the anterior commissure (ac), parietotemporal region at the caudal end of the rf, and parietooccipital region at the posterior commissure (pc). ***D***, Bar graphs for cerebral cortical volume. ***E***, Bar graphs for FO-length. ***F***, Bar graphs for mean cortical thickness. ***G***, Bar graphs for the cortical surface area. ***H***, Bar graphs for global-GI. Data are shown as mean ± SD. Individual data points on ***D*** to ***H*** are overlaid. as, ansinate sulcus; cns, coronal sulcus; crs, cruciate sulcus; csss, caudal suprasylvian sulcaus; FO-length, fronto-occipital length; GI, gyrification index; ls, lateral sulcus; prs, presylvian sulcus; pss, pseudosylvian sulcus; rsss, rostral suprasylvian sulcus; ss, splenial sulcus.

Three-dimensional–rendered images of the dorsal and lateral (left-side) surfaces of the cerebral cortex in LPS-exposed and control ferrets on P20 are shown in Figure 3, *A* and *B*. When coronal (axial) MR images were compared at identical landmark structures of the cerebrum between the two groups of ferrets, the primary sulci extending across the dorsal cortical surface shifted anteriorly in the LPS-exposed ferrets (Fig. 3*C*). The cns and ls extended continuously along the dorsal cortical surface (Fig. 3*A*,*B*), and their boundaries were defined by the ramification of the ansinate sulcus (as) at the level of the caudal end of the rhinal fissure (rf) on the coronal MR images in control ferrets (Fig. 3*C*). The as ramification was observed more anteriorly in LPS-exposed ferrets compared with controls. Additionally, the ls was visible on the dorsal cortical surface at the level of the anterior commissure on the coronal MR images (Fig. 3*C*). In contrast, no differences were observed between LPS-exposed and control ferrets in cerebral cortical morphology measurements, including the cortical volume (*p* = 0.410; Fig. 3*D*), FO-length (*p* = 0.365; Fig. 3*E*), mean cortical thickness (*p* = 0.241; Fig. 3*F*), cortical surface area (*p* = 0.368; Fig. 3*G*), and global-GI (*p* = 0.288; Fig. 3*H*) by Student's *t* test. In addition, the volumes of other brain regions such as the hippocampus and cerebellum differed between the two groups (Table 2).

**Table 2.** Volumes of the hippocampus and cerebellum in 20-d-old LPS–exposed and control ferrets

	LPS (*n* = 4)	Control (*n* = 4)	*F* test	*t* test
Hippocampus	55.6 ± 6.9	50.1 ± 9.7	*p* = 0.584	*p* = 0.456
Cerebellum	141.3 ± 32.5	162.1 ± 36.9	*p* = 0.843	*p* = 0.410

Mean ± standard deviation.

**Figure 4.**
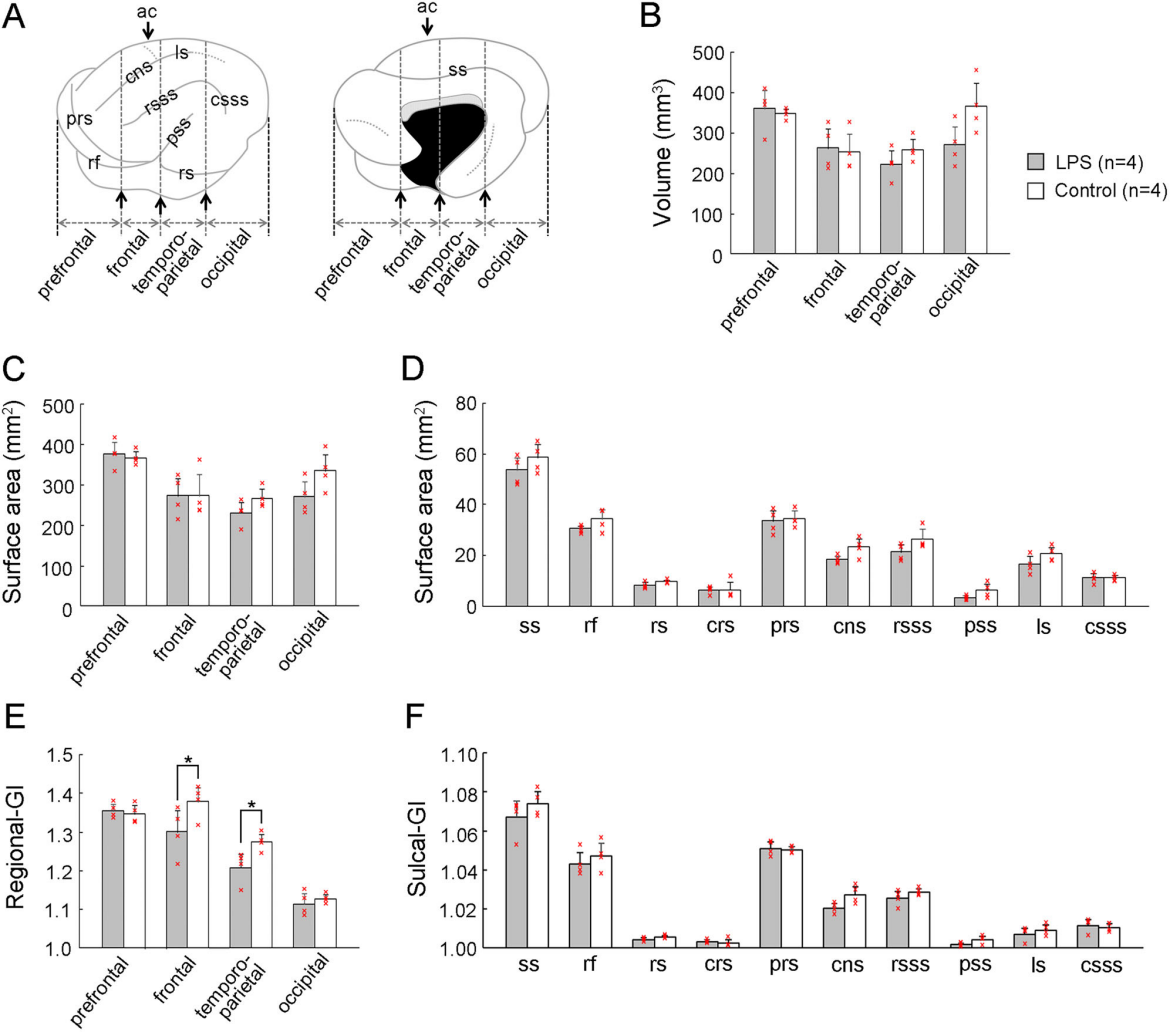
MRI-defined quantitative measurements of four regions of the cerebral cortex. ***A***, Illustrations of cerebral hemispheres at lateral and medial surfaces indicating primary sulci examined and the boundaries of four subdivisions of the cerebral cortex. ***B***, Volumes of four cortical subdivisions. ***C***, Surface areas of four cortical subdivisions. ***D***, Sulcal surface areas. ***E***, Regional-GI of four cortical subdivisions. ***F***, Sulcal-GI. Individual data points on ***B*** to ***F*** are overlaid. *p < 0.001 (Scheffé's test). ac, anterior commissure; cns, coronal sulcus; csss, caudal suprasylvian sulcus; GI, gyrification index; ls, lateral sulcus; prs, presylvian sulcus; pss, pseudosylvian sulcus., rf, rhinal fissure; rs rhinal sulcus; rsss, rostral suprasylvian sulcus; ss, splenial sulcus.

**Figure 5.**
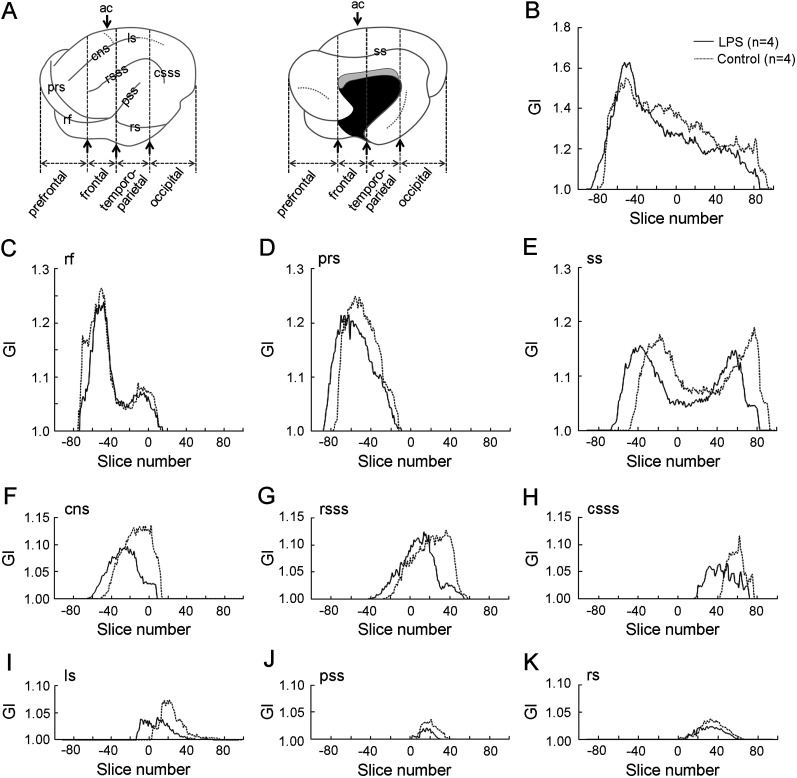
Rostrocaudal GI along the cerebral cortex. ***A***, Illustrations of the cerebral hemispheres showing lateral and medial surfaces indicating primary sulci examined and the boundaries of the four subdivisions of the cerebral cortex. ***B***, Rostrocaudal GI throughout the cerebral cortex. The average GI was estimated for each coronal MR image, and the GI distribution throughout the rostrocaudal axis of the cerebral cortex was represented by aligning the coronal MR image at the level of the anterior commissure as “slice number 0.” ***C***, Sulcal-GI of the rf. ***D***, Sulcal-GI of the prs. ***E***, Sulcal-GI of splenial sulcus (ss). ***F***, Sulcal-GI of the cns. ***G***, Sulcal-GI of the rsss. ***H***, Sulcal-GI of the caudal suprasylvian sulcus (csss). ***I***, Sulcal-GI of the lateral sulcus (ls). ***J***, Sulcal-GI of the pseudosylvian sulcus (pss). ***K***, Sulcal-GI of the rhinal sulcus (rs).

To evaluate regional changes in cerebral cortical morphology following neonatal LPS exposure, the volumes, surface areas, and regional-GIs were estimated in four cortical regions defined by structural landmarks of the cerebrum on coronal MR images, previously described (Sawada et al., 2015). According to our definitions, the primary motor and somatosensory cortices are located within the frontal region (Foxworthy and Meredith, 2011). The temporoparietal region contains the primary auditory and auditory association cortical areas (Keniston et al., 2009) and the parietal cortex (Manger et al., 2002), respectively. The visual cortical area is located in the occipital region (Manger et al., 2002; Homman-Ludiye et al., 2010). The volumes and surface areas of the four regions did not differ between the two groups (Fig. 4*B*,*C*). However, a region-related significant lower regional-GI was detected in the frontal and temporoparietal regions of LPS-exposed ferrets compared with control ferrets (Fig. 4*E*), following a significant effect on an interaction between LPS exposure and cortical regions (*F*_(1,6)_ = 4.917; *p* < 0.05) as revealed by repeated-measure two–way ANOVA. In contrast, the surface areas and sulcal-GIs of all primary sulci examined did not differ between LPS-exposed and control ferrets (Fig. 4*D*,*F*), suggesting no alterations in sulcal infolding by neonatal LPS exposure. A rostrocaudal GI distribution revealed that cortical folding was shifted anteriorly in LPS-exposed ferrets compared with controls (Fig. 5*B*). Notably, the anterior shift of the sulcal infolding was observed in primary sulci located on the medial and dorsolateral surfaces such as splenial sulcus (Fig. 5*E*), cns (Fig. 5*F*), rostral suprasylvian sulcus (rsss; Fig. 5*G*), and ls (Fig. 5*I*). In contrast, the rostrocaudal pattern of sulcal infolding was not altered in the primary sulci located on the ventral cortical surface such as the rf (Fig. 5*C*), pseudosylvian sulcus (Fig. 5*J*), and rhinal sulcus (Fig. 5*K*). These MRI-based morphometric findings revealed that neonatal LPS exposure induced an anterior shift in cortical folding specifically in the medial and dorsolateral regions, without affecting the total volume, surface area, or mean thickness of the cerebral cortex.

### Immunofluorescence staining for various markers with EdU and BrdU labeling

**Figure 6.**
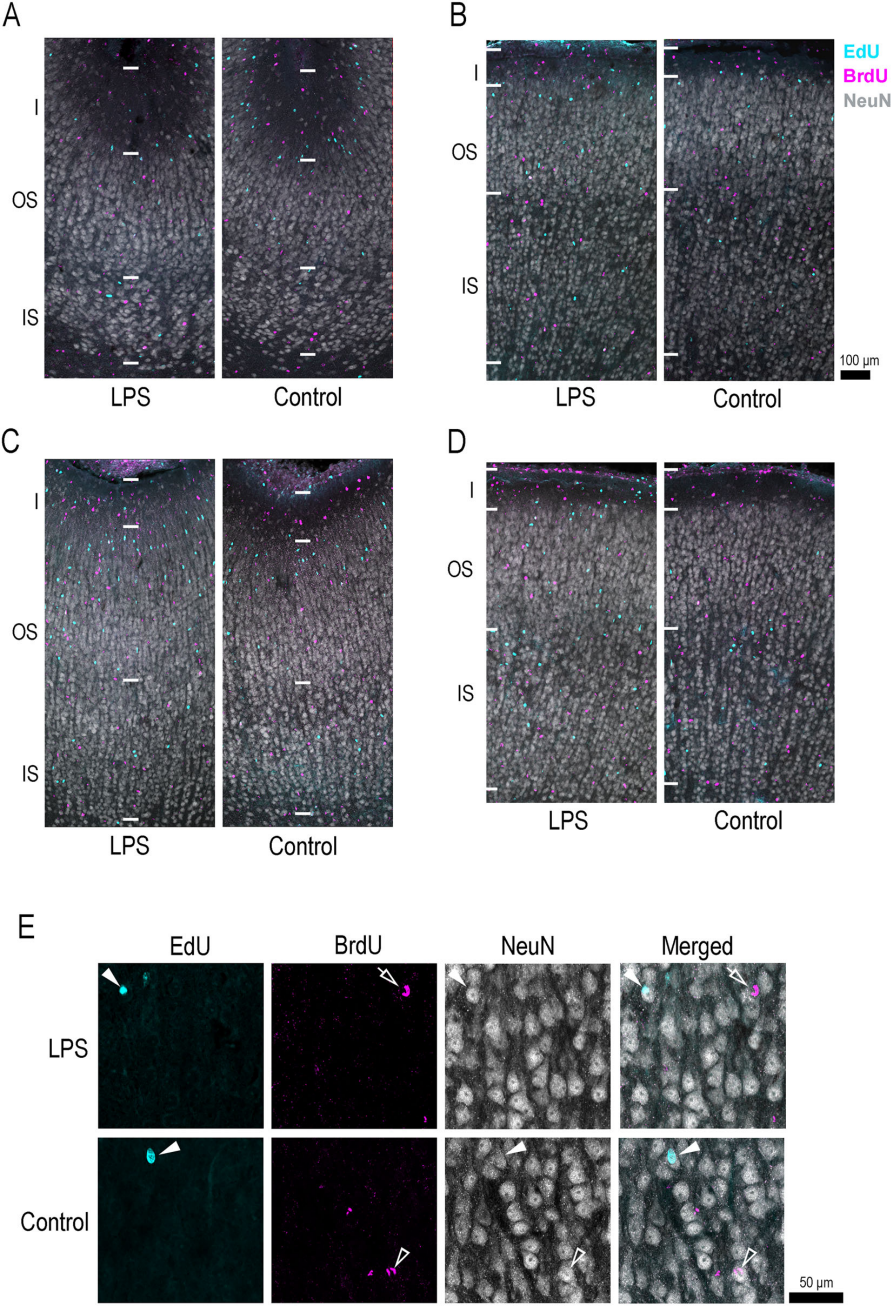
Immunofluorescence for a neuronal marker, NeuN, with EdU and BrdU labeling in representative sulcal and gyral regions of the cerebral cortex in LPS-exposed and control ferrets. ***A***, Low-magnification images of the cortical floor of the cns. ***B***, Low-magnification images of the cortical crown of the CNG. ***C***, Low-magnification images of the cortical floor of the ls, where BrdU-labeled cells were more abundant in LPS-exposed ferrets than in control ferrets. ***D***, Low-magnification images of the cortical crown of the SSG. ***E***, High-magnification images of the OS of the cortical crown of the SSG. BrdU-single labeling was observed less frequently in NeuN immunopositive cells in LPS-exposed ferrets than in control ferrets. Closed arrowheads indicate NeuN immunopositive cells with EdU labeling. Open arrowheads indicate NeuN immunopositive cells with BrdU labeling. Open arrows indicate BrdU-labeled cells. I, Layer I, BrdU, 5-bromo-2′-deoxyuridine; EdU, 5-ethynyl-2′-deoxyuridine, IS, inner stratum.

EdU-labeled cells proliferated on P5, 24 h prior to the first injection of LPS, and BrdU was labeled with cells proliferated on P7 immediately following the second LPS injection. Both EdU+ and BrdU+ cells were distributed throughout the entire depth of the cerebral cortex in both groups of ferrets (Fig. 6*A*–*D*). We estimated the densities of EdU+ and BrdU+ cells in the sulcal floors and gyral crowns of the cortical regions that altered the topology of sulcal infolding (Fig. 5*F*,*I*) and in the prefrontal region, which did not alter sulcal infolding (Fig. 5*D*), in LPS-exposed ferrets. Here, we did not estimate the density of EdU+/BrdU+ cells, which had undergone two rounds of cell division at 48 h intervals. EdU- and BrdU-double labeling was observed in a small population of cells across the cerebral cortex, with densities below 2,000 cells/mm^3^.

**Figure 7.**
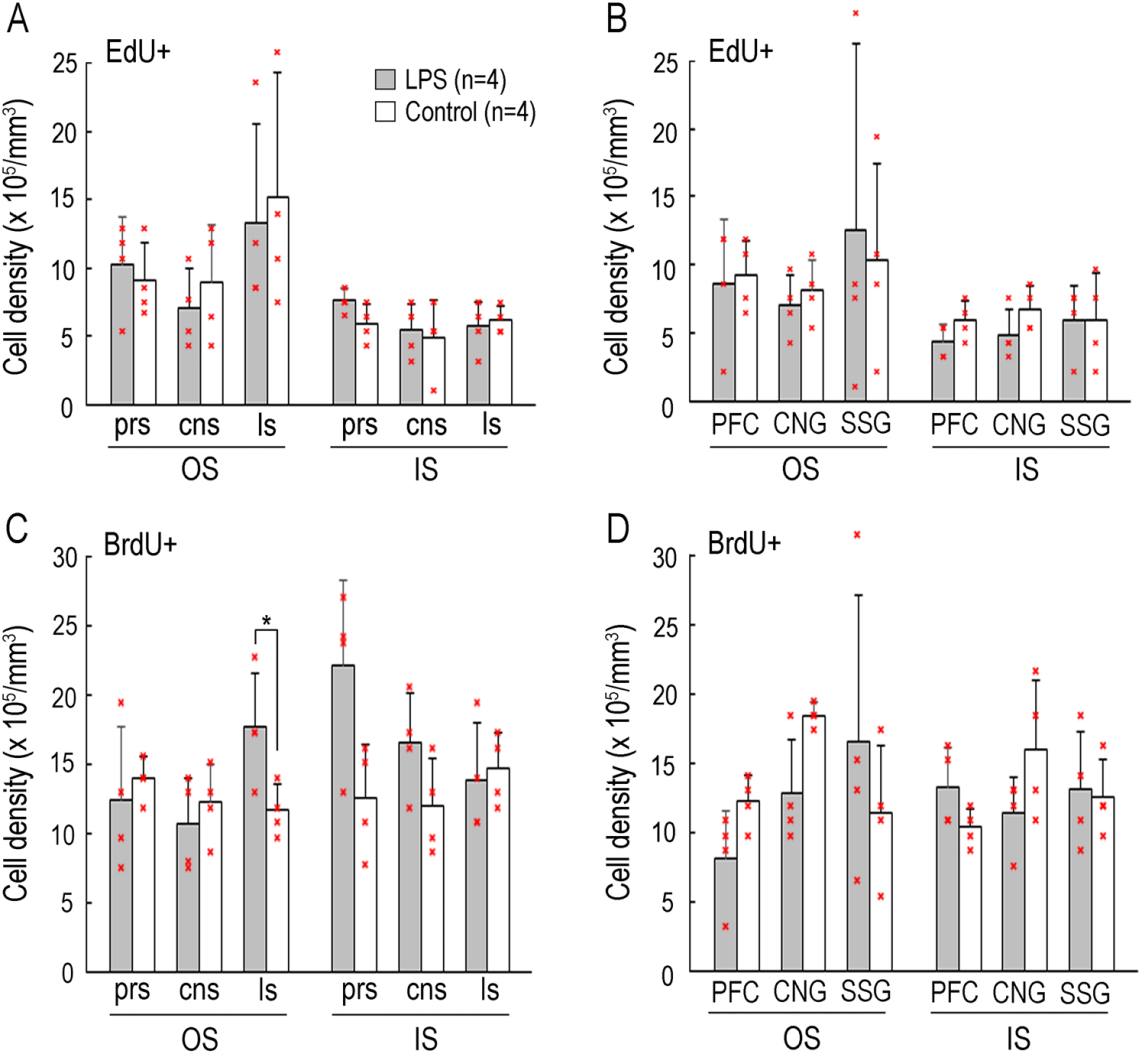
Bar graphs of the densities of EdU- and BrdU-single–labeled cells in representative sulcal and gyral regions. ***A***, Density of EdU-single–labeled cells in the OS and IS of the cortical floors of the prs, cns, and ls. ***B***, Density of EdU-single–labeled cells in the OS and IS of the cortical crowns of the prefrontal gyrus (PFC), CNG, and SSG. ***C***, Density of BrdU-single–labeled cells in the OS and IS of the cortical floors of the prs, cns, and ls. ***D***, Density of BrdU-single–labeled cells in the OS and IS of the cortical crowns of the PFC, CNG, and SSG. Individual data points on ***A*** to ***D*** are overlaid. BrdU, 5-bromo-2′-deoxyuridine; EdU, 5-ethynyl-2′-deoxyuridine.

The density of EdU+ cells did not differ between LPS-exposed and control ferrets in either the OS or IS of any sulcal floors and gyral crowns across the prefrontal (prs and PFC), frontal (cns and CNG), and temporoparietal (ls and SSG) regions (Fig. 7*A*,*B*). However, repeated-measure two–way ANOVA revealed a significant effect on LPS exposure in the BrdU+ cell density in the OS of sulcal floors (*F*_(1,6)_ = 7.028; *p* < 0.05) by Scheffé's post hoc test indicating a significantly higher BrdU+ cell density in the OS of the ls floor in LPS-exposed ferrets compared with controls (Fig. 7*C*). The density of BrdU+ cells did not differ between LPS-exposed and control ferrets in the OS and/or IS of any of the other sulcal and gyral regions examined (Fig. 7*C*,*D*).

### Immunofluorescence staining for NeuN, S100, and cleaved caspase-3 with EdU and BrdU labeling

**Figure 8.**
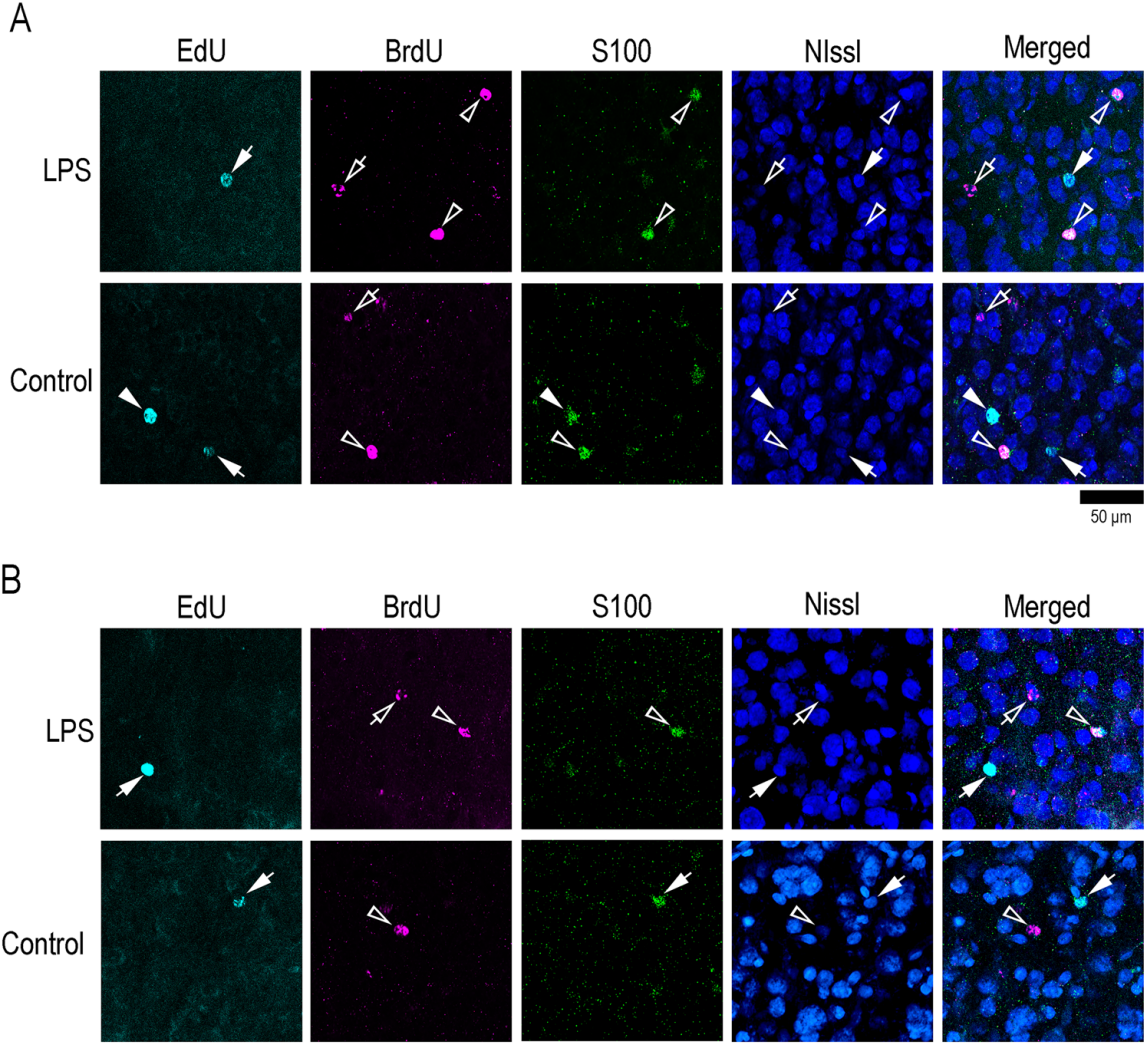
High-magnification images of immunofluorescence for a glial marker, S100, with EdU and BrdU labeling in representative gyral regions of the cerebral cortex in LPS-exposed and control ferrets. The sectional images are counterstained with NeuroTrace 640/660 (Nissl stain). ***A***, OS of the cortical crown of CNG. ***B***, IS of the cortical crown of the CNG. Closed arrowheads indicate S100-immunopositive cells with EdU labeling. Closed arrows indicate EdU-single labeling. Open arrowheads indicate S100-immunopositive cells with BrdU labeling. Open arrows indicate BrdU-labeled cells. BrdU, 5-bromo-2′-deoxyuridine; EdU, 5-ethynyl-2′-deoxyuridine.

**Figure 9.**
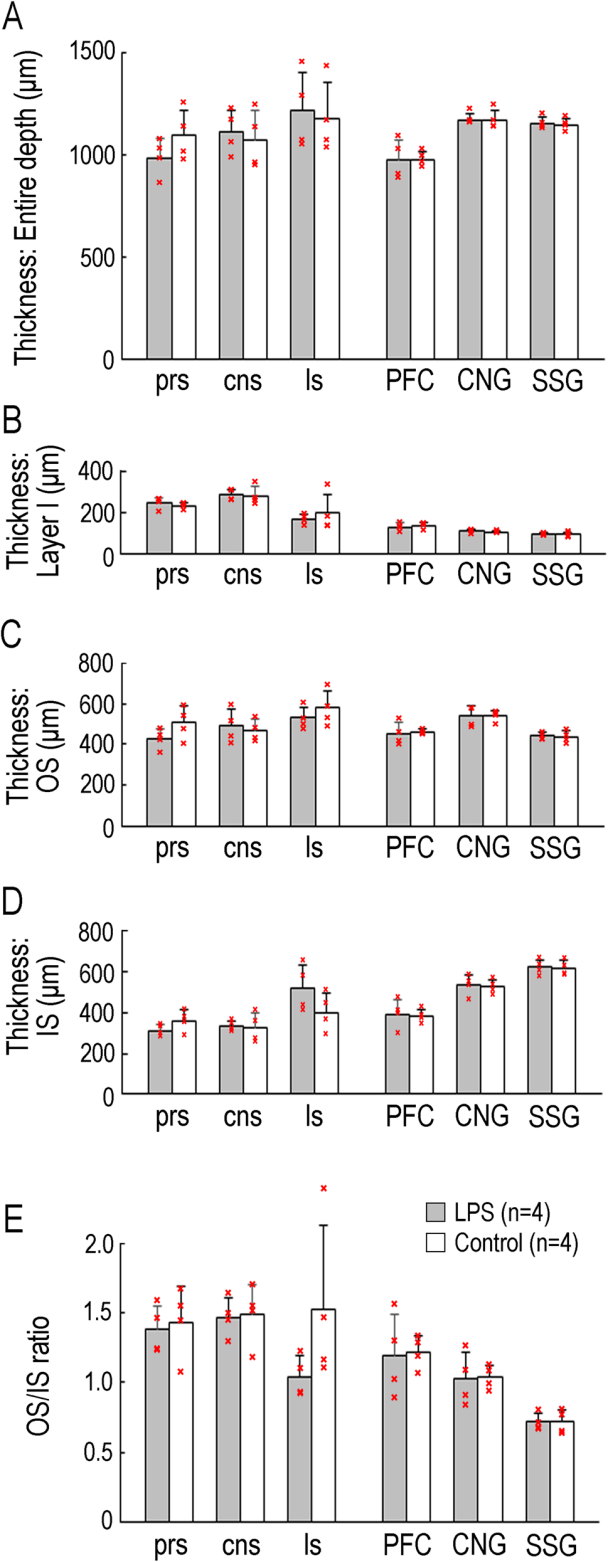
Cortical thickness of representative sulcal and gyral regions of LPS-exposed and control ferrets. ***A***, Thickness of the entire cortex in the cortical floors of the prs, cns, and ls and in the cortical crowns of the prefrontal gyrus (PFC), CNG, and SSG. ***B***, Thickness of Layer I in the cortical floors of the prs, cns, and ls and in the cortical crowns of the PFC, CNG, and SSG. ***C***, Thickness of the OS in the cortical floors of the prs, cns, and ls and in the cortical crowns of the PFC, CNG, and SSG. ***D***, Thickness of the IS in the cortical floors of the prs, cns, and ls and in the cortical crowns of the PFC, CNG, and SSG. ***E***, OS/IS ratio in the cortical floors of the prs, cns, and ls and in the cortical crowns of the PFC, CNG, and SSG. Individual data points on ***A*** to ***E*** are overlaid.

Immunofluorescence staining for NeuN and S100, combined with EdU and BrdU labeling, was performed to determine whether EdU+ and BrdU+ cells differentiated into neurons or glia. Both EdU+ and BrdU+ cells were immunopositive for the neuronal marker, NeuN (Fig. 6), but less immunopositive for the glial marker S100 in either the OS or IS of any sulcal and gyral cortical regions examined (Fig. 8). Laminar structures of the sulcal and gyral cortical regions were clearly observed in the NeuN immunostained sections, and no differences in them were detected between LPS-exposed and control ferrets (Fig. 6*A*–*D*). Since sulcal infolding, as quantified by the sulcal-GI, had been associated with changes in floor cortical thickness of each primary sulci (Sawada et al., 2021), we assessed in the cortical thickness of the prs, cns, and ls floors and the PFC, CNG, and SSG gyral crowns. No significant differences were observed in the thickness of the entire depth of the cerebral cortex, Layer I, OS, or IS between LPS-exposed and control ferrets (Fig. 9*A*–*D*). Furthermore, the OS/IS thickness ratio, which is higher in the sulcal floors and lower in the gyral crowns (Sawada et al., 2021), did not differ between the two groups of ferrets examined (Fig. 9*E*), suggesting that laminar structures of the sulcal and gyral regions were not altered by neonatal LPS exposure.

The percentage of NeuN immunostaining in BrdU+ cells was significantly lower in the OS of the SSG crowns in LPS-exposed ferrets compared with controls but did not differ between the groups in the OS and/or IS of any other sulcal and gyral cortical regions (Tables 3, 4). Thus, neurons born on P7 may have delayed maturation in the SSG. EdU+ cells exhibited <30% S100 immunostaining except in the IS of the cns floors (37.5%) in LPS-exposed ferrets and IS of CNG crowns (37.5%) in control ferrets (Tables 5, 6). Similarly, BrdU+ cells showed <30% S100 immunostaining except in the IS of cns floors (33.3%) and CNG crowns (43.8%) in LPS-exposed ferrets (Tables 5, 6). The proportion of S100 immunostaining did not differ significantly in either EdU+ or BrdU+ cell between LPS-exposed and control ferrets in any of the sulcal and gyral regions examined (Tables 5, 6).

**Table 3.** Percentages of immunostained cells for NeuN in EdU-single- and BrdU-single–labeled cells in sulcus floors of 20-d-old ferrets

	OS		IS	
	LPS	Control	LPS	Control
EdU-single–labeled cells				
prs	70.0% (7/10)	76.9% (10/13)	58.3% (7/12)	62.5% (5/8)
cns	85.7% (6/7)	83.3% (5/6)	60.0% (3/5)	80.0% (4/5)
ls	37.5% (3/8)	64.7% (11/17)	33.3% (2/6)	60.0% (3/5)
BrdU-single–labeled cells				
prs	80.0% (16/20)	76.5% (13/17)	61.3% (19/31)	62.5% (10/16)
cns	73.7% (14/19)	69.2% (9/13)	54.5% (12/22)	70.6% (12/17)
ls	41.4% (12/29)	56.5% (13/23)	46.4% (13/28)	66.7% (12/18)

Percentages were calculated by summing the labeled cells counted within all ROIs from the cerebral hemispheres of four ferrets. The numbers of labeled cells used to calculate percentages are shown in parentheses.

**Table 4.** Percentages of immunostained cells for NeuN in EdU-single- and BrdU-single–labeled cells in gyral crowns of 20-d-old ferrets

	OS		IS	
	LPS	Control	LPS	Control
EdU-single–labeled cells				
PFC	87.5% (7/8)	77.8% (7/9)	60.0% (3/5)	40.0% (2/5)
CNG	90.0% (9/10)	83.3% (10/12)	80.0% (4/5)	66.7% (4/6)
SSG	85.7% (6/7)	80.0% (4/5)	14.3% (1/7)	60.0% (3/5)
BrdU-single–labeled cells				
PFC	75.0% (9/12)	66.7% (10/15)	69.2% (9/13)	72.7% (8/11)
CNG	71.4% (10/14)	56.0% (14/25)	70.6% (12/17)	65.2% (15/23)
SSG	55.2% (16/29)*	84.2% (16/19)	41.2% (7/17)	55.6% (10/18)

Percentages were calculated by summing the labeled cells counted within all ROIs from the cerebral hemispheres of four ferrets. The numbers of labeled cells used to calculate percentages are shown in parentheses.

* *p* *<* 0.05 (*χ*^2^ test).

**Table 5.** Percentages of immunostained cells for S100 in EdU-single- and BrdU-single–labeled cells in sulcus floors of 20-d-old ferrets

	OS		IS	
	LPS	Control	LPS	Control
EdU-single–labeled cells				
prs	0% (0/14)	11.1% (1/9)	0% (0/10)	11.1% (1/9)
cns	0% (0/11)	0% (0/18)	37.5% (3/8)	16.7% (1/6)
ls	0% (0/21)	6.7% (1/15)	28.6% (2/7)	20.0% (1/5)
BrdU-single–labeled cells				
prs	8.3% (1/12)	10.0% (2/20)	10.7% (3/28)	26.7% (4/15)
cns	20.0% (2/10)	15.4% (2/13)	33.3% (6/18)	15.0% (3/20)
ls	5.6% (1/18)	16.7% (2/12)	25.0% (2/8)	20.0% (4/20)

Percentages were calculated by summing the labeled cells counted within all ROIs from the cerebral hemispheres of four ferrets. The numbers of labeled cells used to calculate percentages are shown in parentheses.

**Table 6.** Percentages of immunostained cells for S100 in EdU-single- and BrdU-single–labeled cells in gyral crowns of 20-d-old ferrets

	OS		IS	
	LPS	Control	LPS	Control
EdU-single–labeled cells				
PFC	8.3% (1/12)	16.7% (2/12)	20.0% (1/5)	12.5% (1/8)
CNG	14.3% (1/7)	9.1% (1/11)	14.3% (1/7)	37.5% (3/8)
SSG	0% (0/14)	0% (0/19)	28.6% (2/7)	28.6% (2/7)
BrdU-single–labeled cells				
PFC	10.0% (1/10)	6.3% (1/16)	25.0% (4/16)	23.1% (3/13)
CNG	43.8% (7/16)	25.0% (6/24)	23.1% (3/13)	6.7% (1/15)
SSG	7.7% (1/13)	25.0% (3/12)	36.4% (4/11)	25.0% (3/12)

Percentages were calculated by summing the labeled cells counted within all ROIs from the cerebral hemispheres of four ferrets. The numbers of labeled cells used to calculate percentages are shown in parentheses.

**Figure 10.**
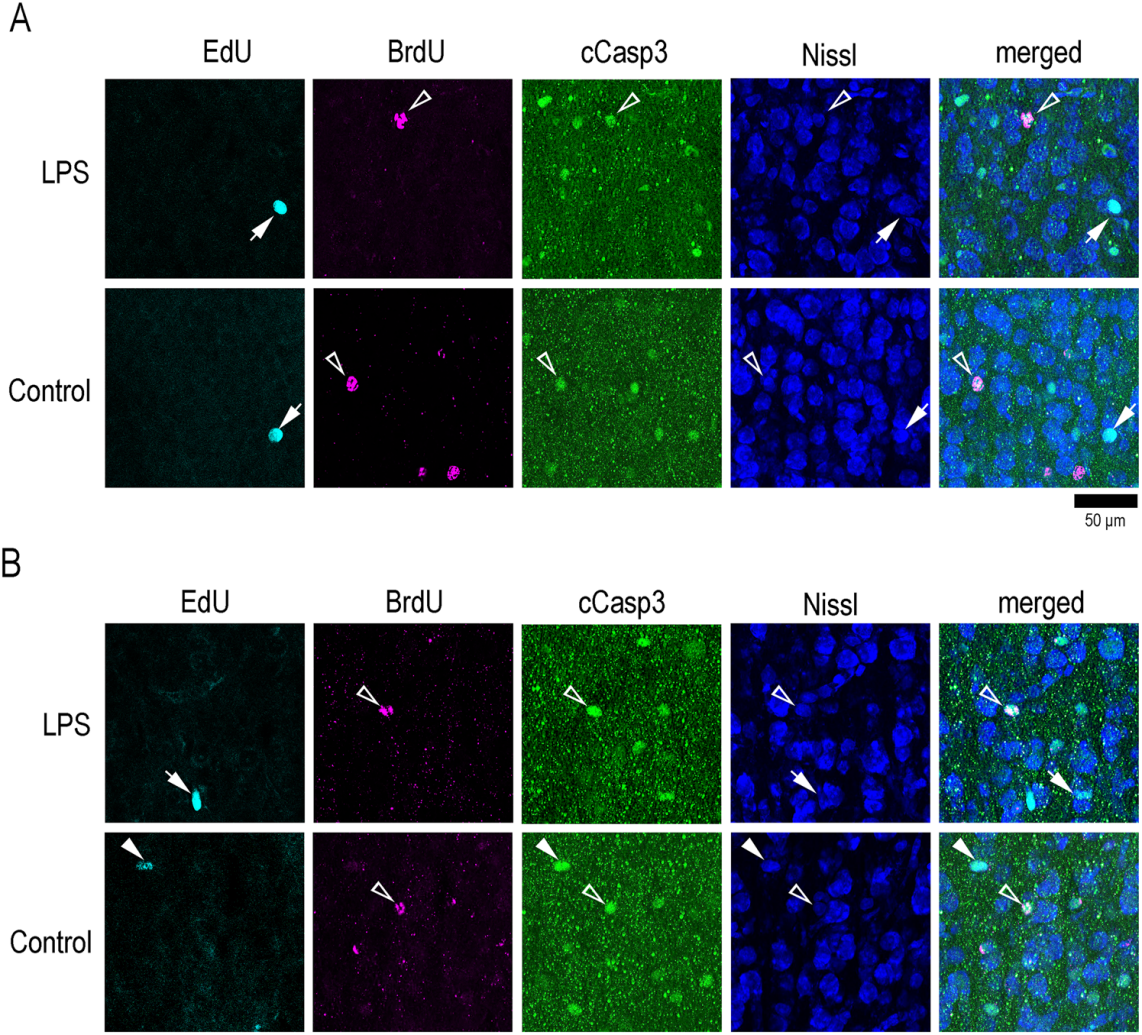
High-magnification images of immunofluorescence for an apoptosis marker, cCaps3, with EdU and BrdU labeling in representative gyral regions of the cerebral cortex in LPS-exposed and control ferrets. The sectional images are counterstained with NeuroTrace 640/660 (Nissl stain). ***A***, OS of the cortical crown of CNG. EdU-single labeling appeared in cCasp3 immunopositive cells less frequently in LPS-exposed ferrets than in control ferrets. ***B***, IS of the cortical crown of the CNG. Closed arrowheads indicate cCasp3 immunopositive cells with EdU labeling. Closed arrows indicate EdU labeling. Open arrowheads indicate cCasp3 immunopositive cells with BrdU labeling. BrdU, 5-bromo-2′-deoxyuridine; EdU, 5-ethynyl-2′-deoxyuridine.

To assess the apoptosis of EdU+ and BrdU+ cells in response to neonatal LPS exposure, we performed immunostaining for cleaved caspase-3 (cCasp3), an apoptotic marker (Gown and Willingham, 2002), in combination with EdU and BrdU labeling. cCasp3+ cells were distributed throughout the cerebral cortex in all sulcal and gyral regions examined in both LPS-exposed and control ferrets (Fig. 10). The proportion of cCasp3 immunostaining in EdU+ cells differed in response to LPS depending on the cortical region. The ratio was significantly higher in LPS-exposed ferrets compared with controls in the OS of the prs and ls floors (Table 7), which are located in the association cortex in the prefrontal and parietal regions, respectively (Manger et al., 2002; Mohapatra and Wagner, 2023). In contrast, the proportion of cCaps3 immunostaining in EdU+ cells was significantly lower in LPS-exposed ferrets than in controls in the IS of the cns floors and in both the OS and IS of the CNG crowns (Fig. 10*A*,*B*; Tables 7, 8), which are situated in the primary motor cortex of the frontal region (Foxworthy and Meredith, 2011). For BrdU+ cells, the cCasp3 immunostaining ratio was particularly high in the OS of the ls floors and CNS crowns (>75.0%) in control ferrets (Tables 7, 8). A significantly lower cCasp3 immunostaining ratio of BrdU+ cells was observed in LPS-exposed ferrets (33.3%) compared with controls (75.0%) that was detected in the OS of the ls floors (Table 7), which are located in the association cortex in the parietal regions (Manger et al., 2002; Mohapatra and Wagner, 2023).

**Table 7.** Percentages of immunostained cells for cCasp3 in EdU-single- and BrdU-single–labeled cells in sulcus floors of 20-d-old ferrets

	OS		IS	
	LPS	Control	LPS	Control
EdU-single–labeled cells				
prs	28.6% (4/14)*	0% (0/13)	33.3% (2/6)	25.0% (1/4)
cns	12.5% (1/8)	22.2% (2/9)	14.3% (1/7)*	71.4% (5/7)
ls	25.0% (5/20)*	4.2% (1/24)	25.0% (2/8)	15.4% (2/13)
BrdU-single–labeled cells				
prs	21.4% (3/14)	28.6% (4/14)	50.0% (11/22)	28.6% (4/14)
cns	30.0% (3/10)	57.9% (11/19)	43.5% (10/23)	28.6% (2/7)
ls	33.3% (6/18)*	75.0% (6/8)	53.3% (8/15)	62.5% (10/16)

Percentages were calculated by summing the labeled cells counted within all ROIs from the cerebral hemispheres of four ferrets. The numbers of labeled cells used to calculate percentages are shown in parentheses.

* *p* *<* 0.05 (*χ*^2^ test).

**Table 8.** Percentages of immunostained cells for cCasp3 in EdU-single- and BrdU-single–labeled cells in gyral crowns of 20-d-old ferrets

	OS		IS	
	LPS	Control	LPS	Control
EdU-single–labeled cells				
PFC	0% (0/9)	7.7% (1/13)	33.3% (2/6)	44.4% (4/9)
CNG	11.1% (1/9)*	57.1% (4/7)	0% (0/6)*	54.5% (6/11)
SSG	15.0% (3/20)	14.3% (2/14)	25.0% (2/8)	20.0% (2/10)
BrdU-single–labeled cells				
PFC	37.5% (3/6)	53.3% (8/15)	27.8% (5/18)	26.7% (4/15)
CNG	58.8% (10/17)	78.9% (15/17)	58.3% (7/12)	42.9% (9/21)
SSG	33.3% (5/15)	45.5% (5/11)	44.4% (8/18)	68.8% (11/16)

Percentages were calculated by summing the labeled cells counted within all ROIs from the cerebral hemispheres of four ferrets. The numbers of labeled cells used to calculate percentages are shown in parentheses.

* *p* *<* 0.05 (*χ*^2^ test).

### Densities of cells immunostained for various marker antigens

**Figure 11.**
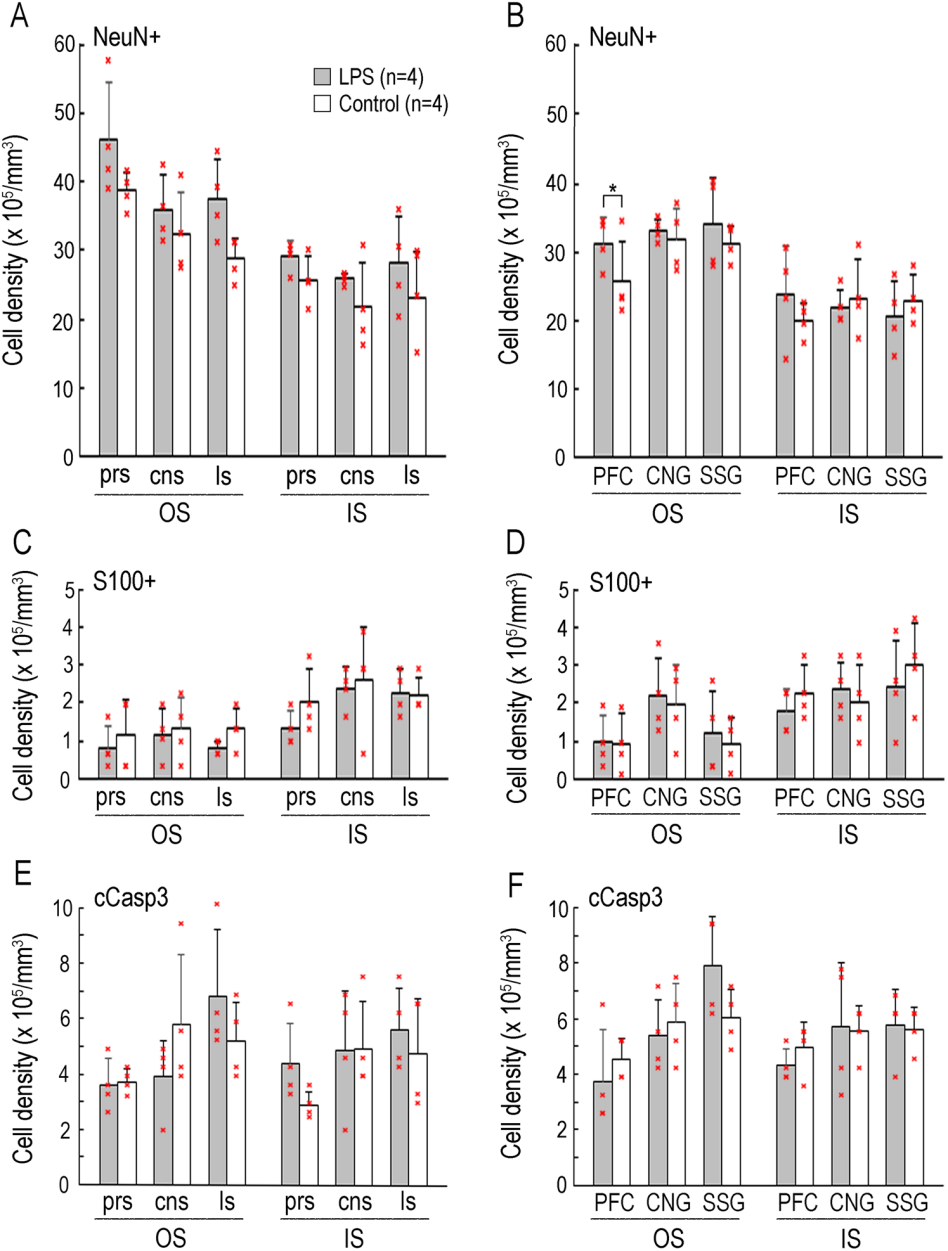
Densities of cells immunostained for various marker antigens in representative sulcal and gyral regions of LPS-exposed and control ferrets. ***A***, Density of NeuN immunopositive cells in the OS and IS of the cortical floors of the prs, cns, and ls. ***B***, Density of NeuN immunopositive cells in the OS and IS of the cortical crowns of the prefrontal gyrus (PFC), CNG, and suprasylvian sulcus (SSG). ***C***, Density of S100-immunopositive cells in the OS and IS of the cortical floors of prs, cns, and ls. ***D***, Density of S100-immunopositive cells in the OS and IS of the cortical crowns of the PFC, CNG, and SSG. ***E***, Density of cCasp3 immunopositive cells in the OS and IS of the cortical floors of prs, cns, and ls. ***F***, Density of cCasp3 immunopositive cells in the OS and IS of the cortical crowns of the PFC, CNG, and SSG. Individual data points on ***A*** to ***F*** are overlaid. *p < 0.01 (Scheffé's test). BrdU, 5-bromo-2′-deoxyuridine; EdU, 5-ethynyl-2′-deoxyuridine.

The total densities of NeuN+, S100+, and cCasp3+ cells were estimated in the OS and IS of the sulcal and gyral regions in both LPS-exposed and control ferrets. Repeated-measure two–way ANOVA revealed a significant effect on LPS exposure (*F*_(1,6)_ = 7.694; *p* < 0.05) in the OS of gyral regions. Scheffé's post hoc test indicated a significantly denser NeuN+ cells in the OS of the PFC (*p* < 0.05; Fig. 11). In contrast, no significant differences in the densities of S100+ and cCasp3+ cells in either the OS or IS of any of the sulcal and gyral regions examined (Fig. 8).

## Discussion

**Figure 12.**
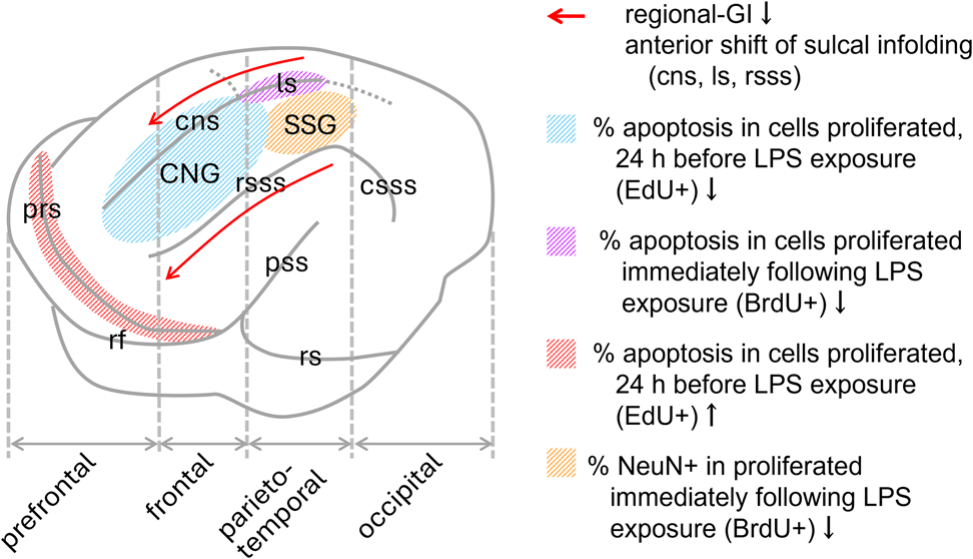
Summary of the MRI-based morphometric and quantitative immunohistochemical findings regarding the effect of neonatal LPS exposure on the cerebral cortex of ferrets. CNG, coronal gyrus; cns, coronal sulcus; csss, caudal suprasylvian sulcus; GI, gyrification index; LPS, lipopolysaccharide; ls, lateral sulcus; prs, presylvian sulcus; pss, pseudosylvian sulcus; rf, rhinal fissure; rs, rhinal sulcus; rsss, rostral suprasylvian sulcus; SSG, suprasylvian sulcus.

The MRI-based morphometric and quantitative immunohistochemical findings from the current study are summarized in Figure 12. An anterior shift in sulcal infolding, including the cns, ls, and rsss, observed in LPS-exposed ferrets is involved in the reduction of sulcal infolding (regional-GI) in the frontal and parietotemporal regions. LPS has dual effects on brain development: a direct effect on neural stem/progenitor cells via TLR4 activation (Okun et al., 2009; Barak et al., 2014) and an indirect effect on differentiating/immature neurons through anti-inflammatory and proinflammatory cytokines released from microglia (Okun et al., 2009; Cowan and Petri, 2018). LPS-mediated activation of TLR4 signaling promotes the proliferation of neural stem/progenitor cells (Grasselli et al., 2018). Our previous study revealed that the proliferation of IPs, followed by the differentiation into Ctip2-expressing postmitotic neurons (differentiative division), was facilitated in the SVZ of ferret infants immediately after LPS exposure (Sawada et al., 2023). In humans, Ctip2 is expressed in postmitotic SVZ progenitors to regulate the expressions of SRGAP1 and ROBO1 to guide axonal outgrowth, independent of their differentiation into Ctip2-expressing cortical projection neurons (Ip et al., 2011). However, neonatal LPS exposure did not influence the density of BrdU-labeled cells on the sulcal floor, except in the ls. LPS-induced differentiative division of IPs through TLR4 activation may not be essential for altered sulcal infolding, as was observed in LPS-exposed ferrets in the present study.

Sulcal infolding can be altered by several factors such as genetics (Kochounov et al., 2010a,b), cortical growth (Toro and Burnod, 2005), relative thickening of the cortical OS or IS (Hilgetag and Barbas, 2005, 2006; Horiuchi-Hirose and Sawada, 2016), and tension in subcortical white matter axons (Armstrong et al., 1991; Van Essen, 1997). Our previous studies have revealed that the proliferation of SVZ progenitors induced by VPA reduces sulcal infolding in the association cortex by cortical thickening in ferrets (Sawada et al., 2021; Sawada, 2022). In contrast, neonatal LPS exposure did not alter cortical thickness, laminar structures, and cortical neuron density. Therefore, factors other than the LPS-mediated proliferative effect on SVZ progenitors may be involved in the altered sulcal infolding in LPS-exposed ferrets.

Programmed cell death is an important mechanism underlying brain morphogenesis and neural circuit formation in the mammalian CNS. A substantial number of neurons are eliminated via apoptosis in the cerebral cortex during normal histogenesis (Roth et al., 2000; Nuñez et al., 2001). Notably, in this study, apoptosis of cortical neurons in ferrets on P20 in response to neonatal LPS exposure varied depending on the cortical region. Apoptosis was enhanced in association cortices of the prefrontal (the prs floors) and parietal (the ls floors) regions but was reduced primarily in the primary motor cortex including the cns floors and the CNG crowns, when postproliferative SVZ progenitors responded to LPS (Fig. 12, Tables 7, 8). LPS-mediated TLR4 activation prevents apoptosis of human neural stem/progenitor cells in vitro (Grasselli et al., 2018). Similarly, apoptosis of postproliferative SVZ and hippocampal dentate gyrus progenitors is reduced immediately following LPS exposure in ferret neonates (Sawada et al., 2023; Sawada and Kamiya, 2025). Conversely, neuronal apoptosis can be induced by proinflammatory cytokines, such as interleukin-6 and tumor necrosis factor-α, released from microglia under immune activations (Shao et al., 2020; Zhou et al., 2021). Therefore, the regional differences in cortical neuronal apoptosis observed in this study may result from a tug-of-war between TLR4-mediated and microglia-mediated LPS effects. This region-related modulation on apoptosis of cortical neurons may be involved in the anterior shift of sulcal infolding in the medial and dorsolateral cortices caused by neonatal LPS exposure.

In the current study, LPS was administered to ferret infants on P6 and P7, but not during the prenatal period. This administration schedule allowed us to investigate the direct effect of LPS on SVZ progenitors, including IPs, which are major sources of cerebral cortical expansion relevant to cortical sulcogyrogenesis (Kelava et al., 2012; Martínez-Cerdeño et al., 2012), independent of MIA. Gyrification abnormalities are a distinct characteristic of human patients with several neurodevelopmental disorders such as ASD and schizophrenia, with phenotypes varying depending on each patient's disorder and sex (Schaer et al., 2013; Wallace et al., 2013; Nenadic et al., 2015; Matsuda and Ohi, 2018; Rafiee et al., 2022; Howes et al., 2023). The pattern of altered sulcal infolding in neonatally LPS-exposed ferrets found in the current findings is distinct from that in ferrets exposed neonatally to VPA, another risk factor for ASD (Sawada et al., 2021). Thus, the use of ferrets as a gyrencephalic animal model will provide novel insights into the pathogenesis of drug- or chemical agent-induced neurodevelopmental disorders including ASD, by elucidating their effects on the proliferation, differentiation, and apoptosis of SVZ progenitors and the resulting diverse sulcal infolding abnormalities.

## Synthesis

Reviewing Editor: Benjamin Deneen, Baylor College of Medicine Decisions are customarily a result of the Reviewing Editor and the peer reviewers coming together and discussing their recommendations until a consensus is reached. When revisions are invited, a fact-based synthesis statement explaining their decision and outlining what is needed to prepare a revision will be listed below. The following reviewer(s) agreed to reveal their identity: NONE. Note: If this manuscript was transferred from JNeurosci and a decision was made to accept the manuscript without peer review, a brief statement to this effect will instead be what is listed below.

The reviewers and I find the work to be of interest and potentially suitable for publication provided substantial revisions. As noted by the reviewers the quality of the immunostaining needs to be drastically improved, as does the quantification of key data as there are concerns about the number of cells included. Notably, NeuN, BrdU, and s100B staining were deemed less than satisfactory and improved imaging will be required. Also, so editing of the abstract and the overall conclusions will be necessary as well, as the reviewers highlight some areas that need clarity and refinement.
